# Continuous low flow ascites drainage through the urinary bladder via the Alfapump system in palliative patients with malignant ascites

**DOI:** 10.1186/s12904-019-0497-3

**Published:** 2019-12-05

**Authors:** Christina Fotopoulou, Thomas Berg, Annekristin Hausen, René Hennig, Rajiv Jalan, Massimo Malagó, Jeroen Capel, Andrea De Gottardi, Guido Stirnimann

**Affiliations:** 1 Imperial College London, Department of Surgery and Cancer, Gynecologic Oncology, Hammersmith Hospital, Du Cane Road, London, W12 0HS UK; 2 Present address: Krankenhaus Freudenstadt, Karl-von-Hahn-Straße 120, 72250 Freudenstadt, Germany; 3 Sektion Hepatologie, Universitätsklinikum Leipzig, Liebigstr. 20, 04103 Leipzig, Germany; 4 Medizinische Klinik und Poliklinik I - Innere Medizin und Gastroenterologie, Universitätsklinikum Bonn, Sigmund-Freud-Str. 25, 53105 Bonn, Germany; 5 Allgemein- und Viszeralchirurgie, Klinikum Stuttgart – Bad Cannstatt, Prießnitzweg 24, 70374 Stuttgart, Germany; 6 Royal Free Hospital, Institute for Liver and Digestive Health, University College London, Rowland Hill Street, London, NW3 2PF UK; 7 Sequana Medical, Technoparkstrasse 1, 8005 Zürich, Switzerland; 8 University Clinic for Visceral Surgery and Medicine, University Hospital Inselspital and University of Bern, Bauchzentrum Bern, 3010 Bern, Switzerland

**Keywords:** Ascites, Pathologic processes, Carcinomatosis, Quality of life, Retrospective studies, Palliative care

## Abstract

**Background:**

Malignant Ascites (MA) is a therapeutic dilemma significantly impairing patients’ quality of life (QoL). The Sequana Medical alfapump System (AP), a subcutaneous, externally rechargeable, implantable device, continually draining ascites via the urinary bladder, has been well established in liver cirrhosis, but not yet in MA. The AP-system was evaluated in cancer patients in reducing the need for large volume paracentesis (LVP).

**Methods:**

A retrospective multicentre evaluation of all eligible patients who received an AP for MA-palliation was performed. AP was evaluated for its ability to reduce LVP and cross-correlated with adverse events (AE), survival and retrospective physician-reported QoL.

**Results:**

Seventeen patients with median age of 63 years (range: 18–81), 70.6% female, across 7 primary tumour types were analysed. Median duration of AP-implantation was 60 min (range: 30–270) and median post-implantation hospital stay: 4 days (range: 2–24). Twelve protocol-defined AE occurred in 5 patients (29.4%): 4 kidney failures, 4 pump/catheter-related blockages, 3 infections/peritonitis and 1 wound dehiscence. Median ascitic volume (AV) pumped daily was 303.6 ml/day (range:5.6–989.3) and median total AV drained was 28 L (range: 1–638.6). Median patient post-AP-survival was 111 days (range:10–715) and median pump survival was 89 days (range: 0–715). Median number of paracenteses was 4 (range: 1–15) per patient pre-implant versus 1 (range: 0–1) post-implant (*p* = 0.005). 71% of patients were reported to have an improvement of at least one physician reported QoL-parameters.

**Conclusions:**

AP appears to be effective in palliating patients with MA by an acceptable morbidity profile. Its broader implementation in oncology services should be further explored.

**Trial registration:**

NCT03200106; June 27, 2017.

## Background

Malignant Ascites (MA) is a common complication of peritoneally disseminated cancers and a therapeutic dilemma significantly impairing affected patients’ quality of life (QoL) [1]. MA often requires repetitive paracenteses to alleviate symptoms such as abdominal distension and discomfort, shortness of breath, and gastrointestinal symptoms [2]. The most common malignancies associated with recurrent MA include ovarian-, breast-, colorectal-, gastric- and unknown primary cancer [2]. The principal aetiology of MA seems to be attributed to the reduced resorption of peritoneal fluid via the peritoneal lymphatic system due to the peritoneal carcinomatosis. Additional potential findings such as liver parenchyma- or porta hepatis metastases aggravate the overall clinical picture [2, 3].

The development of MA is usually a prognostically unfavourable sign with often limited therapeutic options [4]. Management is usually symptomatic including paracentesis and diuretics, while cytotoxic and targeted agents aim at reducing the tumour burden and hence indirectly the production of malignant fluid. In patients with advanced disease and high tumour burden, MA often requires repetitive large volume paracentesis (LVP) which is usually performed under sonographic guidance and has been shown to be effective and feasible in an out- or inpatient setting, depending on the overall clinical picture of the patient [5, 6]. Alternatively, MA may be managed via the placement of a permanent subcutaneous catheter, such as the PleurX, which is an approved treatment modality for MA from various national organisations like the UK based National Institute for Health and Care Excellence (NICE) [7, 8]. Nevertheless, the repetitive puncture and drainage of the peritoneal cavity of patients with disseminated peritoneal carcinomatosis, in addition to being debilitating for the patient, is also associated with significant potential risks such as bowel related complications, port/drain related complications and infections. For these reasons, alternative management options are warranted to minimise risk, alleviate symptoms, and most importantly improve patients’ QoL in this highly palliative situation.

Intra-peritoneal application of the anti-Epcam antibody Catumaxomab had been licensed for MA by the EMA in 2009, however the product has been withdrawn from the US and EU markets in 2013 and 2017, respectively and is not marketed in EU since 2014. Moreover, not all patients were candidates for this treatment, since immunoreaction to the drug often led to an inflammatory response, with pyrexia, nausea, vomiting, abdominal pain and elevated inflammation markers [9].

The Alfapump (AP) System (Sequana Medical, Zürich, Switzerland) is a fully implantable subcutaneous device with a rechargeable battery that moves ascitic fluid from the peritoneal cavity to the urinary bladder from where it is drained by spontaneous diuresis. The AP offers an alternative therapeutic option in terms of a continuous ascitic drainage. This technology was initially developed for cirrhotic patients with refractory ascites and has shown a 90% reduction of ascites with a significant reduction in the requirement of LVP [10]. Due to the completely different pathophysiology, aldosterone antagonists and diuretics do not work, and so novel treatments for MA are of importance. To date, no systematic evaluation of cancer patients with MA treated with the AP is available in the literature; although, a positive case has been reported by Storni et al. [11].

In this retrospective study, we evaluated the performance of the AP in reducing the need for LVP in MA as well as its safety and tolerability.

## Methods

### Study design

This is a retrospective, multicentre study to assess the safety and efficacy of the AP in the palliation of patients with MA. The study involved 6 sites across 3 countries i.e. Germany, Switzerland and the UK. Patients 18 years or older who had undergone an implantation of the AP System for palliative management of their MA between January 2013 and November 2016 were eligible. Patient medical records, implant reports and pump log files were reviewed to obtain data including demographics, history of disease, medical history, pre-implant paracentesis events (number and volume) implant procedure characteristics, post-implant paracentesis/drainage (number and volume), occurrence of medical and technical safety events, antineoplastic treatments and QoL measurements.

The primary endpoint of the study was time to first LVP (defined as therapeutic paracentesis ≥5 L) after AP-implantation. Secondary endpoints included the need of any volume paracentesis, the change in frequency and volume of post-implant LVP compared to the preimplantation period; total ascites volume drained by the AP, device related complications, pump- and patient survival and duration of hospitalisation after the procedure. Adverse events of interest were retrospectively defined and assessed and are stated in full detail in the supplement of this paper (Additional file 1: Table S1). They include obstructive uropathy, ascites leakage defined as a flow of ascitic fluid from the peritoneal cavity to outside the body along the catheter or surgical incisions, or into subcutaneous tissue (early ≤7 days post, late > 7 days post implant), wound dehiscence, pump pocket filled with ascites, not controlled ascites, and pump pocket ulceration.

Exploratory endpoints included subsequent, post-implantation anticancer treatments and assessment of effect of treatment on quality of life (QoL), as retrospectively measured by a physician-assessed questionnaire at the time of patient chart review (Additional file 1: Table S2). As QoL was an exploratory endpoint, the questionnaire was developed by Sequana and was not validated in a patient’s cohort. The treating physician was asked to review the medical records for effect of treatment on the patient’s tiredness, abdominal pain, sleeping, bloating, shortness of breath, appetite, nutritional status and overall status as worsened, no change, improved or indeterminate at the time of collection of the data.

### Device and procedure description

The AP system is a commercially available product consisting of three implantable components: a subcutaneous battery powered pump that is recharged through the skin, a catheter that transfers ascites from the peritoneal cavity into the pump, and a further catheter that pumps the ascites from the AP into the bladder. The AP is fully implanted using minimally invasive surgical techniques and allows normal patient mobility and activity as there are no percutaneous components. The pump battery is charged inductively through the skin using a handheld charger and the AP programmer allows the clinician to wirelessly (through the charger) adapt the system parameters to optimize fluid management according to individual patient needs.

The implantation procedure has previously been described in detail by Stirnimann et al. [10]. In summary, the bladder is being filled with methylene blue-coloured saline solution to facilitate the adequate suprapubic insertion of the AP- bladder catheter. The peritoneal catheter is then tunnelled till the entry point into the abdomen, followed by the subcutaneous positioning of the pump in the right or left upper quadrant below the costal margin.

### Statistical methods

A descriptive analysis was performed. Categorical variables are described using frequencies and percentage of each category. Continuous variables are described using the means, medians, standard deviation or range of each distribution. The analyses were performed in SAS Versions 9.3 or higher (SAS Institute, Cary, USA) and Excel 2016 (Microsoft, Redmond, WA, USA).

Patient survival was calculated by the Kaplan Meier method as days from AP-implantation to patient’s death, or if the patient was still alive, till the date of the last follow up. Pump survival was calculated as days from pump implantation to either device explantation or patients last follow up or death if the pump remained in situ.

### Ethics

The study was approved by the governing ethics committee of each centre and conducted in accordance with ethical and Good Clinical Practice guidelines. An exemption of informed consent was granted for those subjects that were deceased. Informed consent was obtained from those patients that were alive.

## Results

A total of 17 patients were included in the present retrospective analysis. The majority of the patients were female (*n* = 12; 70.6%). Fifteen (88.2%) patients were deceased at the time of the study analysis. The median age at implantation was 63 years (range: 18–81).

Primary malignancy was of hepatic origin in 6 patients (35.3%), epithelial ovarian cancer in 5 patients (29.4%), breast-, uterine-, renal- and pancreatic cancer in 1 patient (5.9%) each and primary cholangiocarcinoma in 2 further patients (11.8%).

Main patients’ comorbidities were cardiovascular (64.7%; *n* = 11), metabolic/nutritional (42.2%; *n* = 7), hepatobiliary (29.4%; *n* = 5), haematologic (17.6%; *n* = 3) and gastrointestinal (17.6%, n = 3). Table 1 summarises the patient demographics and baseline characteristics.

**Table 1:** Demographics and Baseline Characteristics

Characteristic	Result
Demographics	
*Values stated in median and (range)*	
Gender	
Male	5/17 (29.4%)
Female	12/17 (70.6%)
Age at death (years) (*N* = 15);	63 (18–81)
Age at implant (years) (*N* = 17)	63 (18–81)
Height (cm) (*N* = 15)	164 (152–177)
Weight (kg) (*N* = 16)	69 (52–128)
BMI (kg/m^2^) (*N* = 14)	25.5 (18.2–41.3)
Aetiology of ascites: Primary Cancer Type	
Ovarian	5/17 (29.4%)
Breast	1/17 (5.9%)
Uterine	1/17 (5.9%)
Pancreatic	1/17 (5.9%)
Hepatic	6/17 (35.3%)
Cholangiocarcinoma	2/17 (11.8%)
Renal	1/17 (5.9%)
Comorbidities of interest (predefined)	
Cardiovascular disorders^a^	11/17 (64.7%)
Hepatobiliary disorders^b^	5/17 (29.4%)
Haematologic and lymphatic disorders^c^	3/17 (17.6%)
Endocrine disorders^d^	2/17 (11.8%)
Gastrointestinal disorders^e^	3/17 (17.6%)
Metabolic and nutritional disorders^f^	7/17 (41.2%)
Musculoskeletal and connective tissue disorders^g^	2/17 (11.8%)
Nervous system disorders^h^	1/17 (5.9%)
Respiratory, thoracic and mediastinal disorders^i^	1/17 (5.9%)
Skin and subcutaneous tissue disorders^j^	2/17 (11.8%)

^a^ Chronic heart disease [2]; Hypertension [6]; Peripheral arterial disease; Other [3]: Previous pulmonary embolus and cardiac arrest; Pulmonary embolism; Ebstein’s anomaly

^b^ Hepatitis C; Hepatocellular carcinoma, Cholangiocellular carcinoma, Cirrhosis; Hepatic encephalopathy; cirrhosis; fibrolamellar hepatocellular carcinoma

^c^ Idiopathic thrombocytopenic purpura; Progressive oedema; Chronic anemia

^d^ Hypothyroidism; Hypothyreosis

^e^ Duodenal ulcers, esophageal varices grade I, esophageal variceal bleeding; Esophageal varices Grade I/II, portal hypertensive gastropathy; Barrett Oesophagus

^f^ Primary hyperparathyroidism; Diabetes mellitus type II, sarcopenic obesity; Type 2 diabetes [2]; Malnutrition; Hypoalbuminemia; protein deficiency

^g^ Sarcopenia; Bone metastases

^h^ Pseudoradicular syndrome

^i^ COPD, asthma

^j^ Psoriasis

### Procedural characteristics

All procedures were conducted by a minimally invasive approach under general anaesthetic with a median duration of 60 min (range: 30–270) and median post-implantation length of hospital stay of 4 days (range: 2–24). Standard peritoneal catheters were used in 82.4% (14) of cases; the Medionics catheter in 11.8% [2] of cases and in 1 case the catheter specification was not described. 12 (70.6%) patients received routine perioperative antibiotics; six of them (35.3%) were on long-term antibiotic prophylaxis for their underlying condition. Table 2 shows the procedural characteristics in detail.

**Table 2:** Procedural Characteristics

Characteristic	Result
Type of anaesthesia	
General	17/17 (100%)
Median duration of impant procedure in min (range) (*N* = 17)	60 (30–270)
Median length of hospital stay in days (range) (*N* = 17)	4 (2–24)
Prolonged hospitalisation for non-procedure related reasons	6/17 (35.3%)

### Safety

#### Medical adverse events

Thirty days postoperative morbidity included seven adverse events in 4 patients (23.5%): 2 patients presented with an acute kidney injury (AKI) that resolved spontaneously after hydration (one AKI was associated with a sepsis that was rather related to the underlying malignancy than to the AP implantation). One further patient with AKI and liver cancer experienced a variceal bleeding causing death on day 10 post implantation. One patient experienced a wound dehiscence of the left subcostal implantation pocket due to local infection but no signs of systemic infection. The pump pocket was refashioned and the wounds were successfully readapted in local anaesthesia on postop day 10.

In terms of longer-term morbidity, there were 5 complications in 2 patients occurring within the first 3 months and not considered as device deficiencies; 3 of these occurred in 1 patient. Details of all medical adverse events are given in Table 3.

**Table 3:** Medical adverse events – related or unrelated to the AP- implantation (NR: non-resolved)

A				
Patient ID	Adverse event	Days after AP implantation	Resolution day	Day of last follow up or death
AP related				
9	Wound dehiscence of the AP pocket	11	14	111
9	Infection	11	24	111
9	Peritonitis	47	60	111
9	Kidney Failure - Acute on chronic renal failure	2	24	111
11	Kidney Failure - Acute on chronic renal failure	8	NR	10
12	Kidney Failure - KDIGO AKI Stage 2	2	3	562
15	Infection - Sepsis + Acute on Chronic Kidney failure	8	NR	28
AP unrelated				
3	Pleural effusion - chest drain and talc pleurodesis	33	42	81
3	Biliary duct stenosis	60	NR	81
9	Gastrointestinal bleeding	43	43	111
9	Kidney Failure - Acute on chronic renal failure	49	66	111
11	Variceal bleeding	8	Death	10
B				
Patient ID	Event	Days after AP implantation	Resolution day	Day of last follow up or death
Technical issues				
2	Pump blocked and in shake mode	2	8	53 (death)
4	Bladder catheter problem	1	1	45 (death)
8	Pump blocked and in shake mode	18	30	320 (death)
14	Pump problem, humidity problem	344	345	426 (death)

#### Pump- related complications

Pump related complications within the immediate 30 days post-implantation period occurred in 2 patients (11.7%); one pump blockage on days 2 and 18 and one bladder catheter blockage in a further patient, that resolved through reintervention on day 1. In terms of longer-term technical pump-related problems; there was 1 pump failure due to humidity ingress (moisture within the pump electronics) on day 344 that led to a pump exchange. Furthermore, 2 additional surgical reinterventions occurred; 1 explantation of the entire pump (removal of the entire AP-system from the patient) while the patient was still alive (unknown reason) and 1 revision procedure due to blocking of the bladder catheter 1 day after implantation. Data are presented in overview in Table 3.

### Device performance

Eight of the fifteen patients (53.3%) with available data needed regular LVP prior to AP-implantation. Of the 12 subjects with known LVP data post-implant, only one patient required an LVP post AP-implantation and hence there was a clear decrease of the required LVP through the AP. The primary endpoint of time-to-first-LVP was not calculable due to the scarcity of LVP post implant.

Any volume paracentesis was calculable for 15/17 patients pre-implant and 9/17 patients post-implant, with a median number of total paracenteses events of 4 (range; 1–15) per patient pre-implant, and a median of 1 (range; 0–1) events per patient post-implant (*p* = 0.005). Median paracentesis events per 30-day month were 1.30 (range 0.00–4.10; *n* = 15) pre-implant, and 0.08 (range 0–1.2; *n* = 10) post-implant (*p* = 0.059).

The overall volumes removed via paracentesis were evaluable in 10 patients pre-implant with a mean volume of 17.72 ± 12.66 L (median 13.05; range 5.90–44.80) and post-implant in 9 patients (5 no paracentesis) with a mean of 0.96 ± 1.53 L (median 0; range 0.0–4.9).

The overall median volume of ascites drained by the AP was 28 L (range 1.0–638.6; *N* = 17) and the median ascitic volume pumped daily was 303.6 mL/day (range:5.6–989.3, N = 17). Table 4 summarises the volume drained data.

**Table 4:** Comparison of Pre and Post-implant Paracentesis and LVP – Events and Volumes per Patient

	N	Median	Q1	Q3	Mean	Std	Min	Max
Prior to Implant								
Paracentesis^a^								
Event Rate (# Events/Months)	15	1.3	0.52	2.5	1.53	1.222	0	4.1
Volume Rate (Liters/Months)	10	6.78	2.96	12.05	7.89	6.026	1.8	21
Event Count	17	4	2	6	4.41	3.759	1	15
LVP^b^								
Event Rate (# Events/Months)	6	1.93	1.36	3	11.45	23.797	0.5	60
Volume Rate (Liters/Months)	6	12.35	8.64	21	67.43	36.08	5.3	345
Event Count	8	2	1	4	2.5	1.69	1	5
Post Implant								
Paracentesis								
Event Rate (# Events/Months)	10	0.08	0	0.57	0.31	0.458	0	1.2
^c^Volume Rate (Liters/Months)	9	0.28	0	1.13	3.17	6.894	0	21
Event Count	10	1	0	1	0.6	0.516	0	1
LVP								
Event Rate^d^ (# Events/Months)	1	1.07	1.07	1.07	1.07	.	1.1	1.1
^c^Volume Rate (Liters/Months)	1	5.36	5.36	5.36	5.36	.	5.4	5.4
Event Count	1	1	1	1	1	.	1	1

^a^ includes LVP events and volumes. ^b^ Rates based upon observation period from first paracentesis to implant (not first LVP)

^c^Volumes calculated only for patients with known paracentesis events – patients with null events are not included

^d^ Only one patient had an event – no data on frequency, timing, nor volume available

If Paracentesis’s data is missing but LVP’s is not missing, then LVP’s data is used for paracentesis

In summary, only four patients needed an additional post-implant drainage – of any volume- of their ascites.

### Oncology related outcomes, pump survival and QoL

**Fig. 1:**
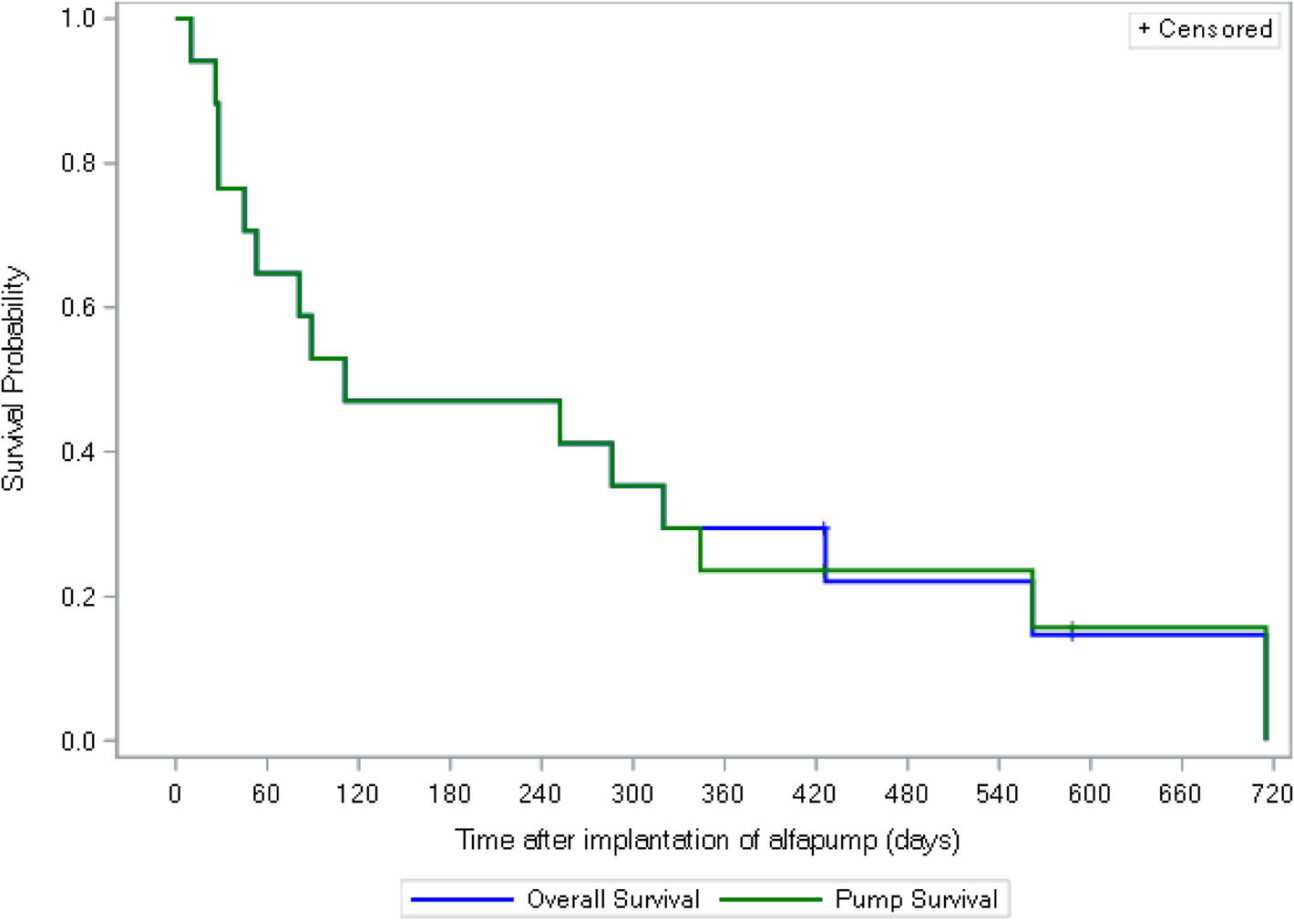
Patient and Pump Survival Kaplan-Meier

Median patient survival was 111 days (range: 10–715; *n* = 17) and median device survival 89 days (range: 0–715; 17/18 pumps) (Fig. 1). A total of 16/17 patients had a pump still in-situ at final outcome of death or end of the present study; in one patient the pump had to be explanted.

Eleven patients received systemic and anticancer treatment after AP-implantation within a median time of 31 days post implantation (range: 2–253 days). Three patients received palliative care alone, without any further anticancer treatment. In 3 patients no exact information was available on their further oncologic management.

Retrospective treating physician assessment of effect of treatment on quality of life revealed that 8 of 14 (57.1%) evaluable patients had an improved overall QoL in the post-implant period, in 3 (23%) there was no change, only 2/14 (14.3%) patients were considered to have a reduced QoL and one patient could not be assessed for overall status. More details of the QoL scores with respect to individual domains are given in Table 5. In three patients no retrospective QoL analysis could be performed due to lack of data.

**Table 5:** Retrospective QoL assessment by the treating physician (chart review)

Characteristics	Worsened	No change	Improved	No information
How did the alfapump affect the patient’s:				
Tiredness	2/14 (14.3%)	5/14 (35.7%)	5/14 (35.7%)	2/14 (14.3%)
Abdominal pain	1/14 (7.1%)	2/14 (14.3%)	10/14 (71.4%)	1/14 (7.1%)
Sleeping	1/14 (7.1%)	3/14 (21.4%)	4/14 (28.6%)	6/14 (42.9%)
Bloating	1/14 (7.1%)	0/14 (0%)	8/14 (57.1%)	5/14 (35.7%)
Shortness of breath	1/14 (7.1%)	4/14 (28.6%)	7/14 (50.0%)	2/14 (14.3%)
Appetite	2/14 (14.3%)	6/14 (42.9%)	5/14 (35.7%)	1/14 (7.1%)
Nutritional status	3/14 (21.4%)	6/14 (42.9%)	3/14 (21.4%)	2/14 (14.3%)
Overall status	2/14 (14.3%)	3/14 (21.4%)	8/14 (57.1%)	1/14 (7.1%)

## Discussion

The present study represents the first systematic evaluation of the AP- device for the palliative management of MA across patients with various epithelial tumour types. We could demonstrate a significant reduction of the requirements for any volume and large volume paracenteses through the AP compared to the preimplantation period. The surgical morbidity profile was acceptable considering the high-risk profile of the treated patients. Only four patients needed an additional post-implant drainage of their ascites through an extra catheter. Moreover, as per the evaluation of the treating physicians, 71% of the patients experienced an improvement in at least one domain of QoL such as tiredness, abdominal bloating and pain, sleeping, shortness of breath, appetite and nutritional status. A comparison of patient survival between gynae and gastrointestinal cancers would have provided a meaningful analysis, however, due to the very small number of total patients, no separate valid analysis for each cancer type could be performed.

These results encourage the consideration of the AP device also outside the area of cirrhosis related ascites, into the field of palliative cancer medicine, even though further prospective studies are warranted. The safety and efficacy data we have demonstrated in the present evaluation are similar to the ones already established for cirrhotic patients. The experience on 56 cirrhotic patients in a post market surveillance registry study demonstrated within a median follow-up period of 5.8 months a median reduction in post-implant paracentesis rate from 2.17 to 0.17 events per month [12]. Additionally, 44 device and procedure related safety events were experienced, compared to 4 events in 4 (23.5%) patients in the present study. Surgical reintervention was required in 17 (21.4%) patients in the cirrhosis - replacement of pump is not included - and in 3 (17.6%) patients in the present study.

Nevertheless, the repetitive puncture and drainage of the peritoneal cavity of patients with disseminated peritoneal carcinomatosis, in addition to being debilitating for the patient, is also associated with significant potential risks such as bowel related complications, port/drain related complications and infections. Further issues such as loculation of the ascites result in ineffective palliative drainage on the long term. Even though our experience is mainly with PleurX, as the main approved treatment modality for MA in the UK, there are numerous other manufacturers across the various countries around Europe. Their common feature is that they are semi-permanent, are being inserted radiologically via usually sonographic guidance and hence the risk of visceral injury, especially in patients after extensive peritoneal surgery with dense adhesions, is increased. Furthermore, in immunosuppressed cancer patients, such external systems that are being exposed to constant manipulation via the patients themselves or health personnel, are potentially associated with higher risk of infections, blockages and malfunction of the external part of the catheter, that makes its clinical application often challenging. Therefore, a closed system, without any direct communication to external exposures has potentially a lower risk of infection. Also, since it is being inserted under direct vision, the risk of direct visceral injury is lower.

As we confirmed in the present analysis, acute kidney injury is a perioperative challenge that needs addressing. For that reason, in a way forward, robust protocols would need to be set in place to adequately replace volume, during and immediately after the anaesthetic procedure, under the perspective of a proactive management. Since the patients are anyway hospitalized for a few days post implantation, this fluid replacement can occur under controlled circumstances in a safe environment even in this highly palliative setting.

Anecdotally, on the example of gynae cancer patients alone, they did not report in clinical practice any pollakisuria (higher frequency of micturition) or dysuria that would additionally negatively affect their QoL; just reported higher volumes of urine released during voiding. Nevertheless, this could not be not retrospectively assessed and would need to be evaluated in any future prospective study.

The present analysis has, nevertheless, significant shortcomings which need to be considered when interpreting the data and therefore future prospective studies are warranted to resolve these issues and answer still open questions. The retrospective design on a multicentre level had as a consequence the lack of completeness of the requested data, especially in a population that has in its majority deceased. This applies especially in the QoL evaluation, which was just a retrospective estimation of the treating physicians and hence requires further exploration. The small sample, especially per cancer type, led to only assumptive hypothesis regarding the value of the pump on all patients with MA and therefore more targeted and focussed studies need to be designed to resolve the issues, challenges and effectiveness of the pump in particular cancer types. A further drawback is the lack of cost estimates in relation to the standard treatment. Future studies will need to focus on the costing’s comparison of the relatively pricey pump compared to the simple ascitic drainage and whether this is being adequately counterbalanced with the gain from the reduced hospitalisation times, potential positive effect on electrolyte disbalances, nutritional status of the treated patients etc.

Even though our analysis has all these limitations, it still may set the basis for a prospective multicentre evaluation with the view of a broader potential implementation of the AP in oncology services. In cancer patients with liver cirrhosis, the pathophysiology of ascites production may differ from patients with a peritoneal carcinosis but preserved liver function. However, this has not been specifically addressed in this retrospective study. There is a prospective trial in planning with the AP, so that more robust and valid data can be generated.

A significant aspect that needs to be addressed and represents a potential concern of treating oncologists, is the risk of implantation of malignant cells in the urinary bladder mucosa with development of metastatic lesions or even secondary cancers and the risk of recurrent urinary tract infections (UTI) and stone formation/encrustation. With an average patient survival of only 3.5 months, and in view of the anyway diffusely disseminated disease, longer term events like a stone formation or bladder cancerous lesions are rather clinically non-relevant, even though that is at the present moment a theoretical assumption. In the ovarian cancer cohort, the majority of the patients underwent a diagnostic cystoscopy in the first 4–6 weeks post AP- implantation and none of the patients showed bladder mucosal abnormalities. No bladder biopsy was performed if the mucosa was macroscopically normal, since any microscopic mucosal infiltration was without clinical significance or consequences. In the designing of a future prospective study, routine cystoscopies should be considered to establish and confirm these initial observations. Nevertheless, in this highly palliative setting with limited patient survival, secondary microscopic bladder wall seeding is relative and has to be put in context with the overall clinical picture of a diffuse peritoneal dissemination.

A further and potentially promising aspect of the AP technology is the prospective of forming the basis of a translational platform with a non-invasive, continuous and biomolecular profiling in epithelial cancers during antineoplastic treatment. Through the tumour cell harvest from the urine we could theoretically have a continuous, non-invasive, access to a liquid biopsy of the patients that could be used for drug resistance testing and mutational profile analysis [13]. How realistic and feasible such an approach would be, needs to be addressed in future studies.

## Conclusion

We could demonstrate in this retrospective analysis, that AP appears to be relatively effective and safe in palliating patients with MA, while seemingly improving their QoL. Its implantation is rather straightforward and minimally invasive, resulting in reduced necessity of repetitive ascitic drainages, so that its broader implementation in oncology services should be further explored in prospective and comparative clinical trials to establish its value. A prospective trial is currently in planning to compare standard practice techniques with the AP including comparative analysis of cost effectiveness, prospective evaluation of QoL, impact on patients nutritional and metabolic status and overall wellbeing.

## Supplementary information

**Additional file 1: Table S1.** Includes the adverse events of interest defined by the protocol of the study. **Table S2.** consists of the questions and possible answers in the Quality of Life Questionnaire used in the study.

## Data Availability

The data that support the findings of this study are available from Sequana Medical, but restrictions apply to the availability of these data, which were used under license for the current study, and so are not publicly available. Data are however available from the authors upon reasonable request and with permission of Sequana Medical.

## Abbreviations

AE: Adverse event
AKI: Acute kidney injury
AP: Alfapump
AV: Ascitic volume
LVP: Large volume paracentesis
MA: Malignant ascites
QoL: Quality of life
