# Supplementary material for: Continuous low flow ascites drainage through the urinary bladder via the Alfapump system in palliative patients with malignant ascites
Source: BMC Palliat Care. 2019 Dec 5;18:109. doi: 10.1186/s12904-019-0497-3 (PMC6896754; doi:10.1186/s12904-019-0497-3)
Supplement: Supplementary file 1 — Additional file 1: Table S1. Includes the adverse events of interest defined by the protocol of the study. Table S2. consists of the questions and possible answers in the Quality of Life Questionnaire used in the study. [file 12904_2019_497_MOESM1_ESM.docx]

# Supplementary Tables

| **Table S1.** Protocol-defined Complications of Interest |
| --- |
| Complications of Interest |
| - Infection |
| - Kidney Failure |
| - Obstructive uropathy |
| - Ascites leakage – early (≤7 days post implant) |
| - Ascites leakage – late (>7 days post implant) |
| - Wound dehiscence |
| - Pump pocket filled with ascites |
| - Ascites not controlled |
| - Pump pocket ulceration |
| - Device-related complications |
| - Acute kidney injury |
|  |

Supplementary table 1 includes the adverse events of interest defined by the protocol of the study.

Supplementary table 2 consists of the questions and possible answers in the Quality of Life Questionnaire used in the study.

| **Table S 2.** Physician-assessed Quality of Life Questionnaire | |
| --- | --- |
| **Question** | **Answer** |
| How did the alfapump affect the patient’s tiredness? | Worsened  No change  Improved  No information |
| How did the alfapump affect the patient’s abdominal pain? |  |
| How did the alfapump affect the patient’s sleeping? |  |
| How did the alfapump affect the patient’s bloating? |  |
| How did the alfapump affect the patient’s shortness of breath? |  |
| How did the alfapump affect the patient’s appetite? |  |
| How did the alfapump affect the patient’s nutritional status? |  |
| How did the alfapump affect the patient’s overall status? |  |
|  | |
